# The *aceE* involves in mycolic acid synthesis and biofilm formation in *Mycobacterium smegmatis*

**DOI:** 10.1186/s12866-020-01940-2

**Published:** 2020-08-18

**Authors:** Suting Chen, Tianlu Teng, Shuan Wen, Tingting Zhang, Hairong Huang

**Affiliations:** National Clinical Laboratory on Tuberculosis, Beijing Key laboratory for Drug Resistant Tuberculosis Research, Beijing Chest Hospital, Capital Medical University, Beijing Tuberculosis and Thoracic Tumor Institute, Beijing, 101149 China

**Keywords:** *Mycobacterium smegmatis*, *aceE*, Biofilm, Mycolic acid, Cell wall

## Abstract

**Background:**

The integrity of cell wall structure is highly significant for the in vivo survival of mycobacteria. We hypothesized that changes in morphology may indicate changes in cell wall metabolism and identified an *aceE* gene mutant (*aceE*-mut) which presented a deficient colony morphology on 7H10 agar by screening transposon mutagenesis in *Mycolicibacterium smegmatis*, basonym *Mycobacterium smegmatis* (*M. smegmatis*). This study aimed to identify the functional role of *aceE* gene in cell wall biosynthesis in *M. smegmatis*.

**Results:**

We observed that the colony morphology of *aceE*-mut was quite different, smaller and smoother on the solid culture medium than the wild-type (WT) strain during the transposon library screening of *M. smegmatis*. Notably, in contrast with the WT, which aggregates and forms biofilm, the *aceE*-mut lost its ability of growing aggregately and biofilm formation, which are two very important features of mycobacteria. The morphological changes in the *aceE*-mut strain were further confirmed by electron microscopy which indicated smoother and thinner cell envelope images in contrast with the rough morphology of WT strains. Additionally, the *aceE*-mut was more fragile to acidic stress and exhibited a pronounced defects in entering the macrophages as compared to the WT. The analysis of mycolic acid (MA) using LC-MS indicated deficiency of alpha-MA and epoxy-MA in *aceE*-mut strain whereas complementation of the *aceE*-mut with a wild-type *aceE* gene restored the composition of MA.

**Conclusions:**

Over all, this study indicates that *aceE* gene plays a significant role in the mycolic acid synthesis and affects the colony morphology, biofilm formation of *M. smegmatis* and bacteria invasion of macrophage.

## Background

The mycobacterial cell wall is mainly composed of three types of macromolecules i.e. peptidoglycan, arabino-galactan and mycolic acids (specific components shared by the members of the order Corynebacterineae (e.g., mycobacteria, nocardia, and rhodococci)) [1]. Glycolipids, porins and lipoarabinomannan as well as its variants, which are anchored to the cell membrane by diacylglycerol, are also essential components [2]. The carboxyl group of mycolic acids is vertically covalently linked to the hydroxyl group of arabino-galactan by ester bond, arabino-galactan is linked to the peptidoglycan layer by phospholipid bond, whereas other glycolipids and free lipids are regularly distributed in the thicker layer of mycolic acids [3–5]. These collectively form a thick, dense, poorly permeable cell wall, which not only allows mycobacteria to resist the dry environment and harmful chemicals but also allows it to reproduce in the macrophages [4, 6]. Therefore, the molecules involved in the cell wall biosynthesis of TB bacilli have been considered as important anti-TB drug targets. Among the existing anti-TB drugs, isoniazid, ethionamide and ethambutol target the cell wall synthesis pathways, among which isoniazid and ethambutol are the first-line anti-TB drugs.

Pyruvate dehydrogenase (PDH) is an enzyme complex that catalyzes the conversion of pyruvate into acetyl-coA in vivo. The complex mainly consists of three enzymes, which are respectively called E1, E2 and E3 components of PDH according to the order in which they participate in the reactions. Through a series of chemical reactions of pyruvate decarboxylation, the glycolytic pathway (the final product is pyruvate) and the tricarboxylic acid cycle (the initial reactant is acetyl-coA) can be effectively connected [7]. As an important intermediate metabolite, acetyl-coA not only participates in tricarboxylic acid cycle as well as the glyoxylate cycle but also provides carbon source for the synthesis of mycolic acid and lipids [8, 9]. Studies have also found that the genes involved in gluconeogenic pathway and glyoxylate cycle are up-regulated in Mtb isolated from macrophages, mouse lung tissues and tissue samples of patients, suggesting that the compensatory metabolism of acetyl-coA is necessary for intracellular growth and persistence in vivo [10, 11].

In the present study, we found a mutant strain in which the *aceE* gene, encoding the E1 component of PDH, was inactivated by Himar1 transposon insertion (*aceE*-mut). This mutant had obvious differences in colony morphology (smaller plaque, edge smooth and round) and defects in biofilm formation in contrast with the wild-type (WT) strains. Further analyses indicated that the *aceE* gene deficiency affected the cell mycolic acid profile of *M. smegmatis*.

## Results

### The *aceE*-Mut exhibited unusual colony morphology

**Fig. 1:**
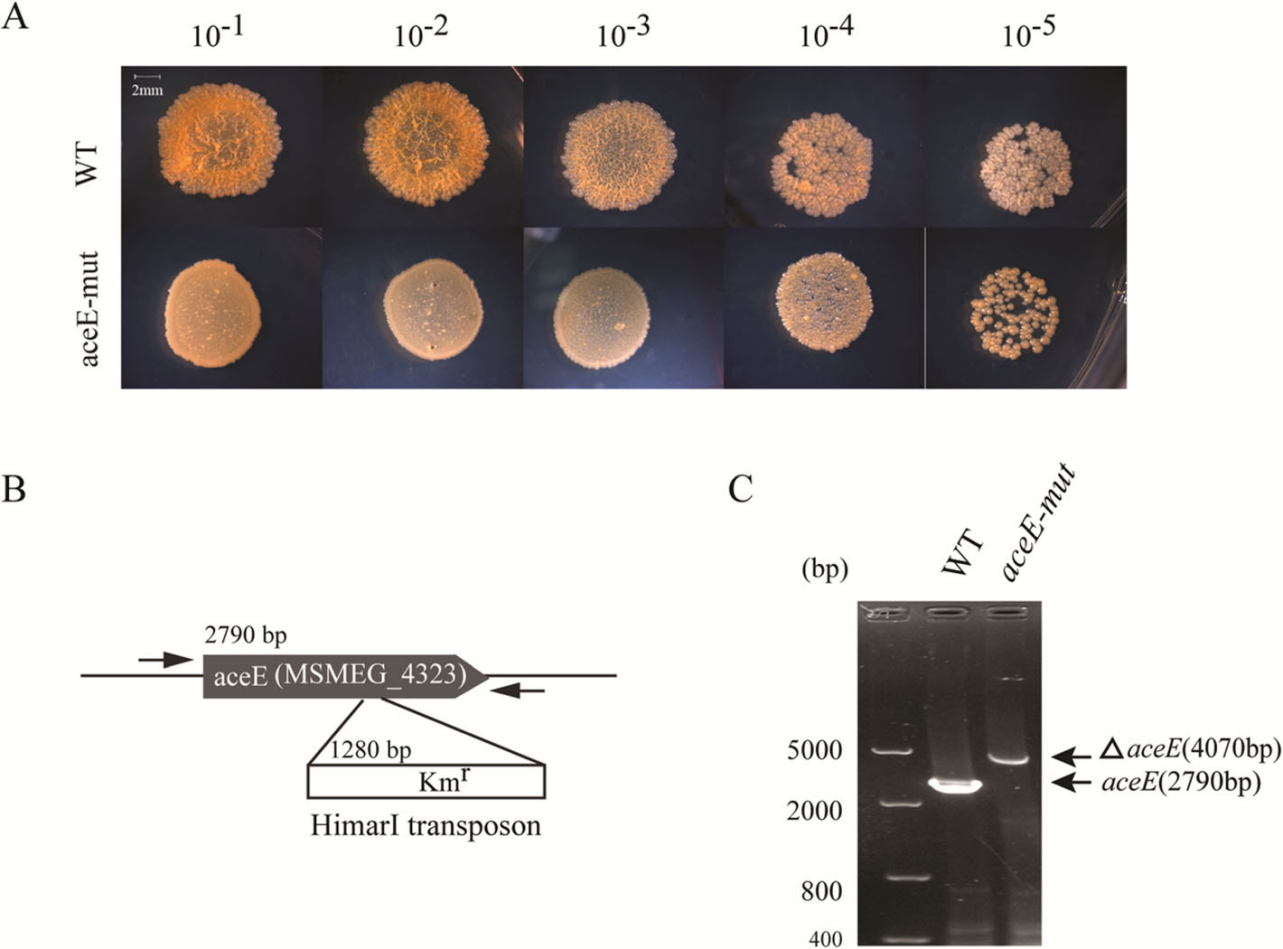
The identification of *M. smegmatis aceE*-mut. **a**. The *aceE*-mut showed smoother colony morphology in contrast to WT. The culture of *M. smegmatis* mc^2^155 and *aceE*-mut were 10× diluted and 10 μL culture aliquots were spotted on the Middlebrook 7H10 medium supplemented with 0.2% glycerol. The images were taken after incubation at 37 °C for 3 days on 7H10 plates. **b**. HimarI transposon insertion site in *aceE* gene; **c**. PCR verification of the *aceE* transposon mutant. WT: *M. smegmatis* mc^2^155; *aceE*-mut: *aceE* gene deficiency mutant selected from *M. smegmatis* mc^2^155 transposon library

**Fig. 2:**
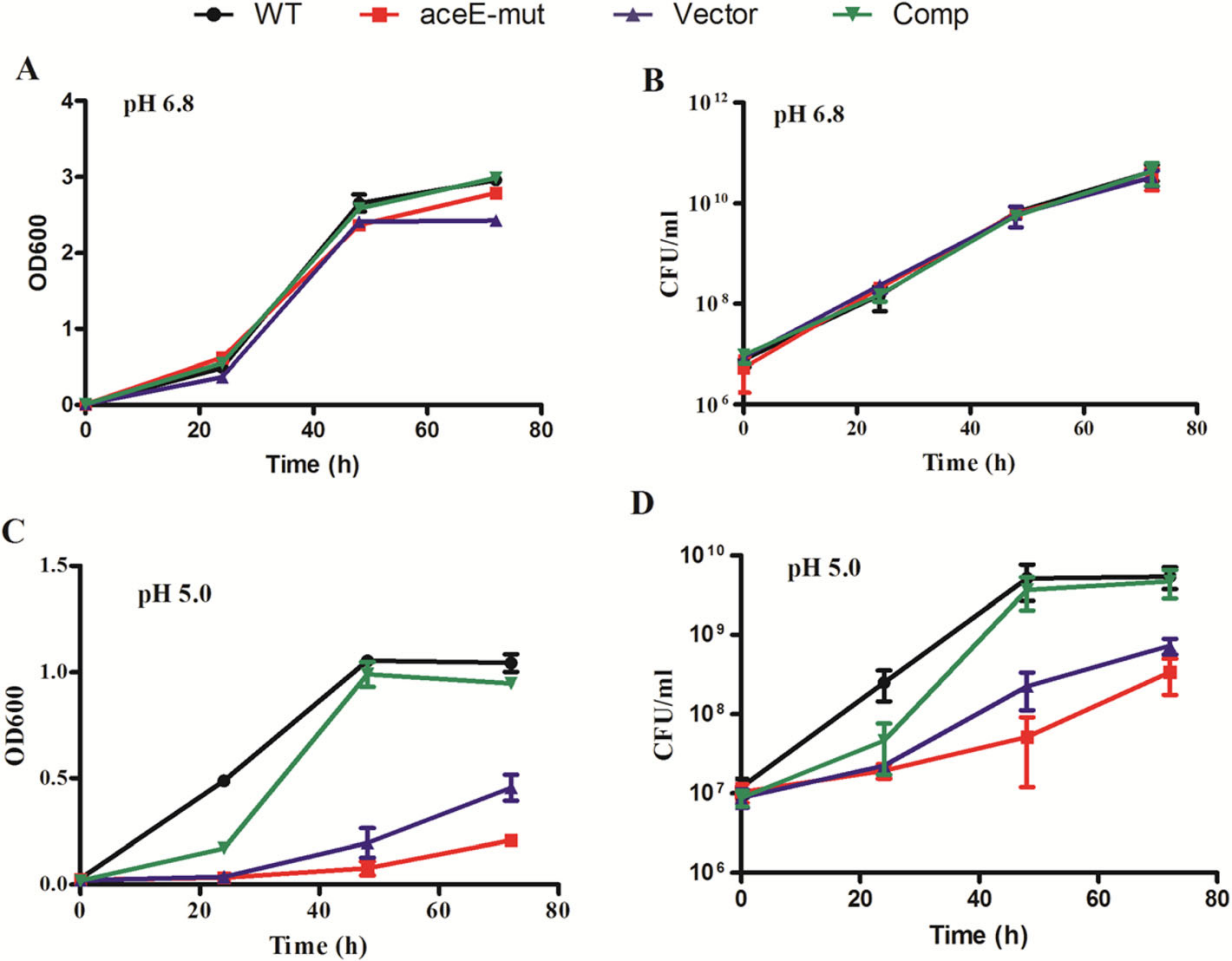
The *aceE*-mut strain was sensitive to acid stress. **a** and **b**. Bacterial strains were grown in the neutral Middlebrook 7H9 medium supplemented with 0.05% Tween-80 and 0.2% glycerol; **c** and **d**. Bacterial strains were grown in the acified Middlebrook 7H9 medium (pH 5.0) supplemented with 0.05% Tween-80 and 0.2% glycerol. The OD_600_ and CFU were determined at an interval of 24 h. The graph is a representation of one of three independent experiments. The mean ± SD of triplicate experimental samples is shown from one experiment, Error bars represent SD

By screening the *M. smegmatis* transposon library, a transposon mutant showed obvious differences in colony morphology (small, smooth without jagged edges and yellow color) on agar plate when compared to the parental WT *M. smegmatis* strain (Fig. 1a). DNA sequencing and analysis of the MycoMar/*M. smegmatis* chromosomal junction revealed that the transposon mutant had an insertion at a TA dinucleotide within the *aceE* gene and the distance of the Tn insertion from the start codon of the *aceE* gene is 2243 bp (Fig. 1b and c). No apparent difference in growth rate was found between the WT and *aceE*-mut strain with the Middlebrook 7H9 neutral medium culturing (Fig. 2a and b). However, we observed that the *aceE*-mut strain dispersed uniformly in the broth without Tween-80, while the WT *M. smegmatis* formed more clumps in the same medium. Notably, the growth rate of *aceE*-mut was significantly lower than the WT strain in acidified 7H9 media during logarithmic and stationary phases (Fig. 2c and d). Comp strain demonstrated similar growth dynamics as WT in both culture conditions. These results demonstrated that *aceE* gene is dispensable in *M. smegmatis* and the disruption of *aceE* gene in *M. smegmatis* renders the bacteria more sensitive to acid stress, and this feature could be complemented with the wild-type copy of *aceE* gene.

### Pellicle and biofilm formation defected in *aceE*-Mut

**Fig. 3:**
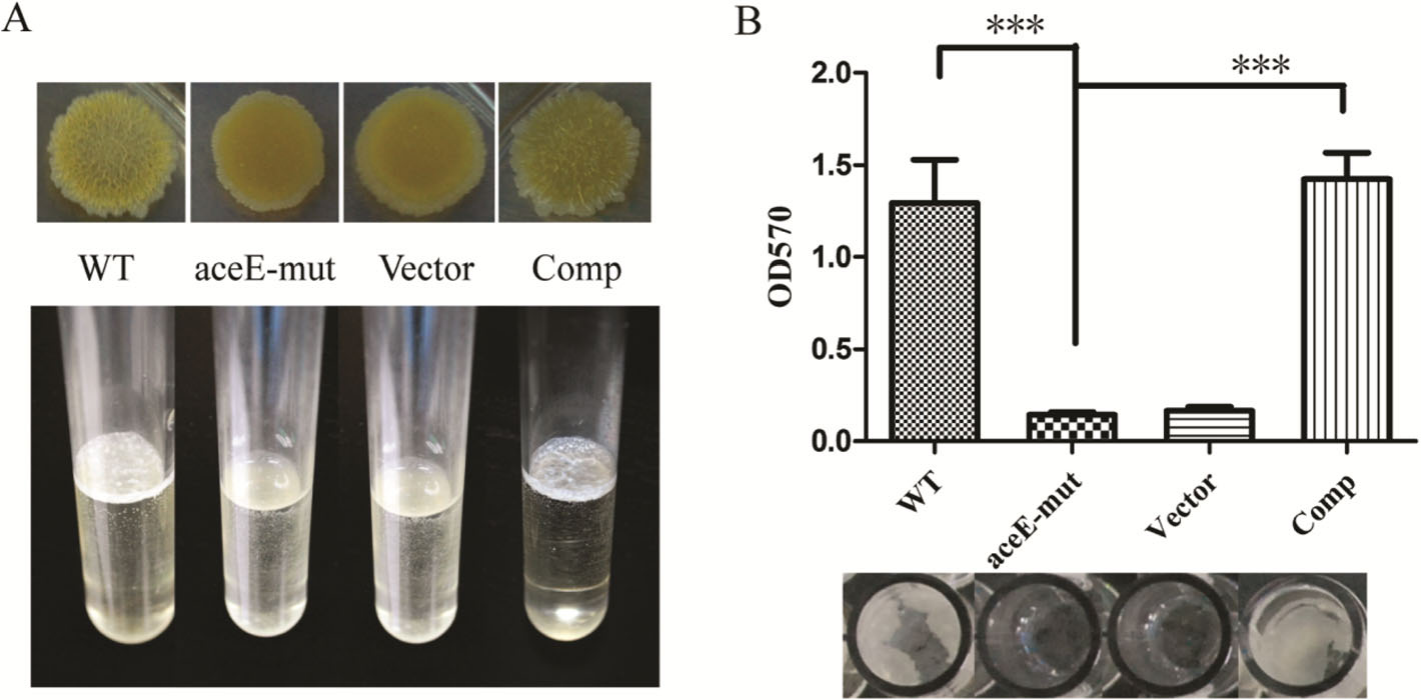
The effect of *aceE* deficiency on pellicle and biofilm formation of mycobacteria. **a**. *aceE*-mut is defective in pellicle formation, while the complementary expression of *aceE* gene in *aceE*-mut stain can recover the formation of pellicle in 7H9 medium without Tween-80 in standing culture. Each experiment performed in triplicate. **b**. Quantification of the biofilm formation after crystal violet staining. Mean optical density for five biological replicates per strain ± SD for a representative experiment from 3 experiments is shown. Significant differences were determined by One Way ANOVA and are indicated by *** (*P* < 0.001). Error bars represent SD

In contrast to the significant pellicle growth that appeared on the air-liquid interface in the WT strain culture, pellicle was absent from the *aceE*-mut strain culture when grown in 7H9 medium without Tween-80 supplement and shaking (Fig. 3a). In order to better quantify the biofilm formation defects in *aceE*-mut, strains were cultivated in M63-based liquid medium and biofilm formation was analyzed visibly as well as using the crystal violet assay. Consistently, *ace*E-mut did not form biofilm in the M63-based liquid medium either. Notably, the complementary expression of *aceE* gene in *aceE*-mut strain restored the pellicle and biofilm formation to the WT levels (Fig. 3b). Taken together, these results indicated that *aceE* gene is involved in pellicle and biofilm formations.

### *aceE* gene affected the cell surface morphology and cell wall architecture of *M. smegmatis*

**Fig. 4:**
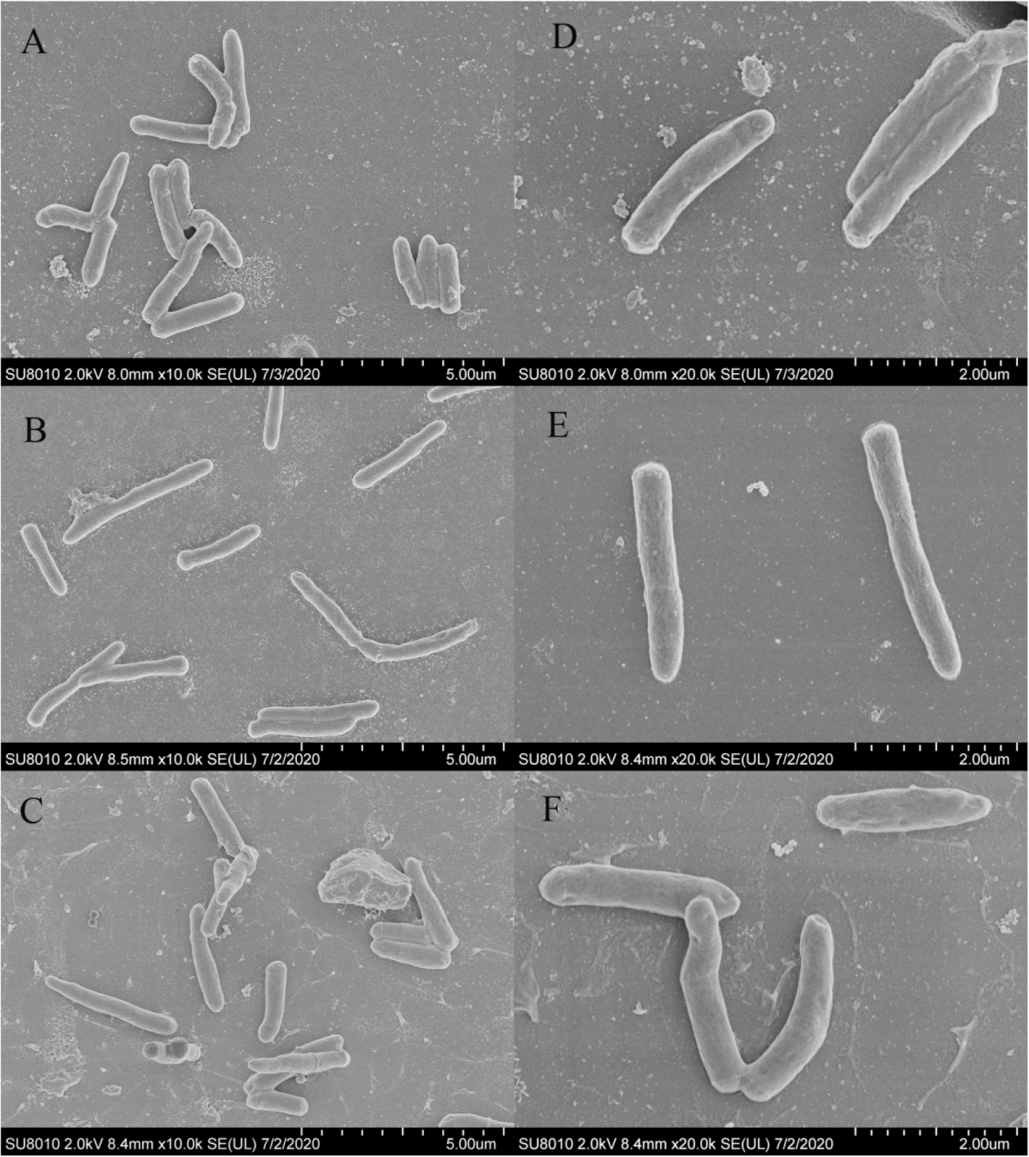
The morphology of *M. smegmatis* mc^2^155 (**a** and **d**), *aceE*-mut (**b** and **e**) and Comp (**c** and **f**) under SEM. Bars represent 5 μm (**a**, **b** and **c**) and 2 μm (**d**, **e** and **f**)

**Fig. 5:**
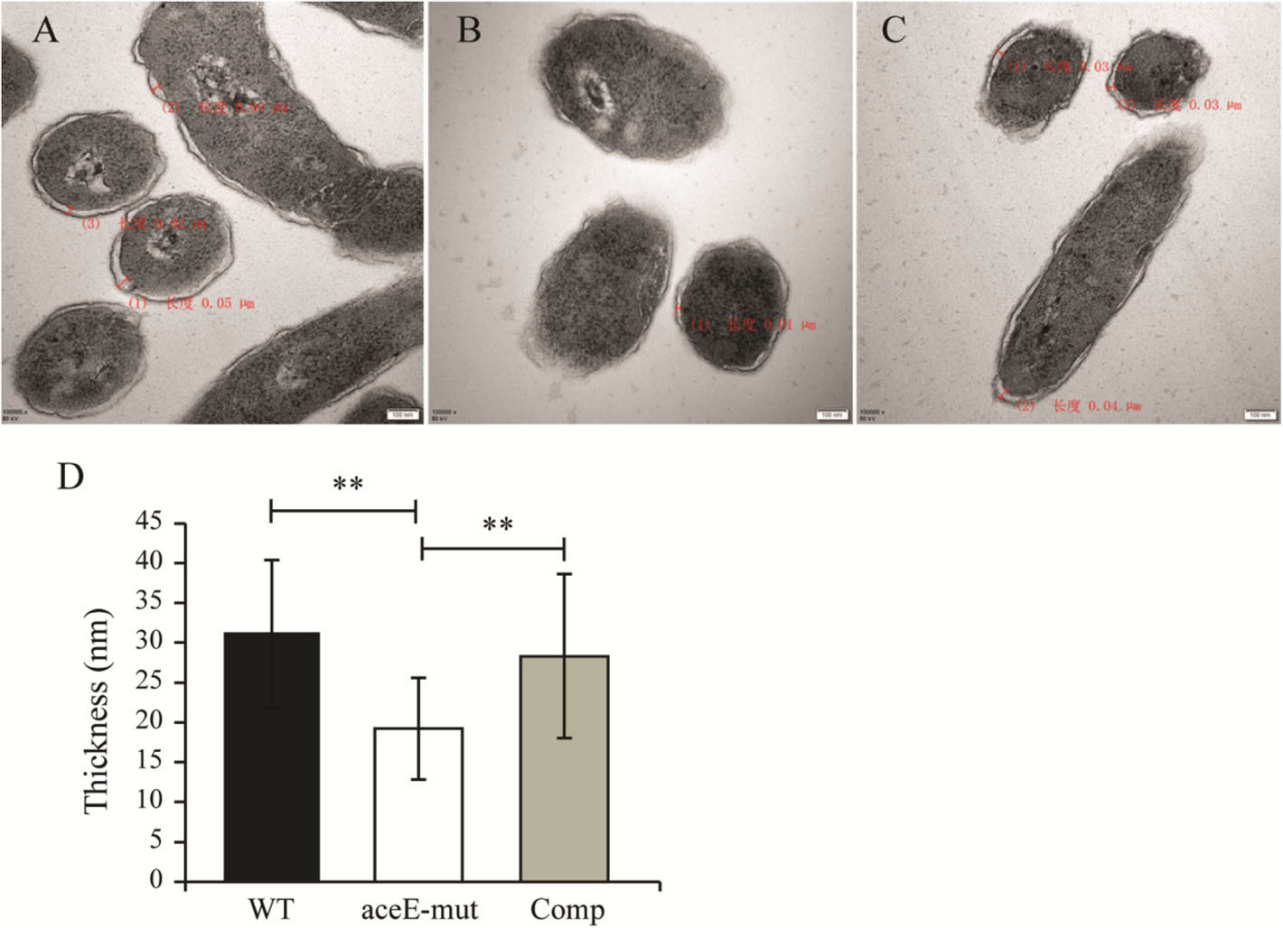
The cell wall thickness of *M. smegmatis* mc^2^155 (**a**), *aceE*-mut (**b**) and Comp (**c**). D. The quantification of the cell wall thickness for each strain, one hundred mycobacteria in the visual field were randomly selected. The largest distance between the outer membrane and the inner membrane of each cell was measured and statistically analyzed. Bars represent 100 nm (**a**, **b** and **c**). Histogram bars in panel (**d**) indicate standard deviation. Significant differences were determined by One Way ANOVA and are indicated by ** (*P* < 0.01)

The surface morphology of WT and *aceE*-mut was observed by scanning electron microscopy. The *aceE*-mut cells were slightly slender than WT cells, and their surfaces were smoother than WT cells (Fig. 4). These microscopic observations were consistent with the smooth phenotype on agar plate. The cell morphology and cell wall architecture were further examined by transmission electron microscopy, which showed that the cell wall of the mutant was thinner (*P* < 0.01) than WT and Comp strains (Fig. 5).

### Cell wall permeability analysis

In order to analyze the effect of disruption of the *aceE* gene on cellular response against stress conditions in vitro, the *aceE*-mut and WT strains were treated with commonly used anti-TB drugs and several antimicrobial agents in vitro. No significant differences in antimicrobial sensitivity tests between WT and mutant strains were observed (Supplementary Table S1 and Supplementary Fig. S1).

### aceE-Mut has a defect in invasion of macrophages

**Fig. 6:**
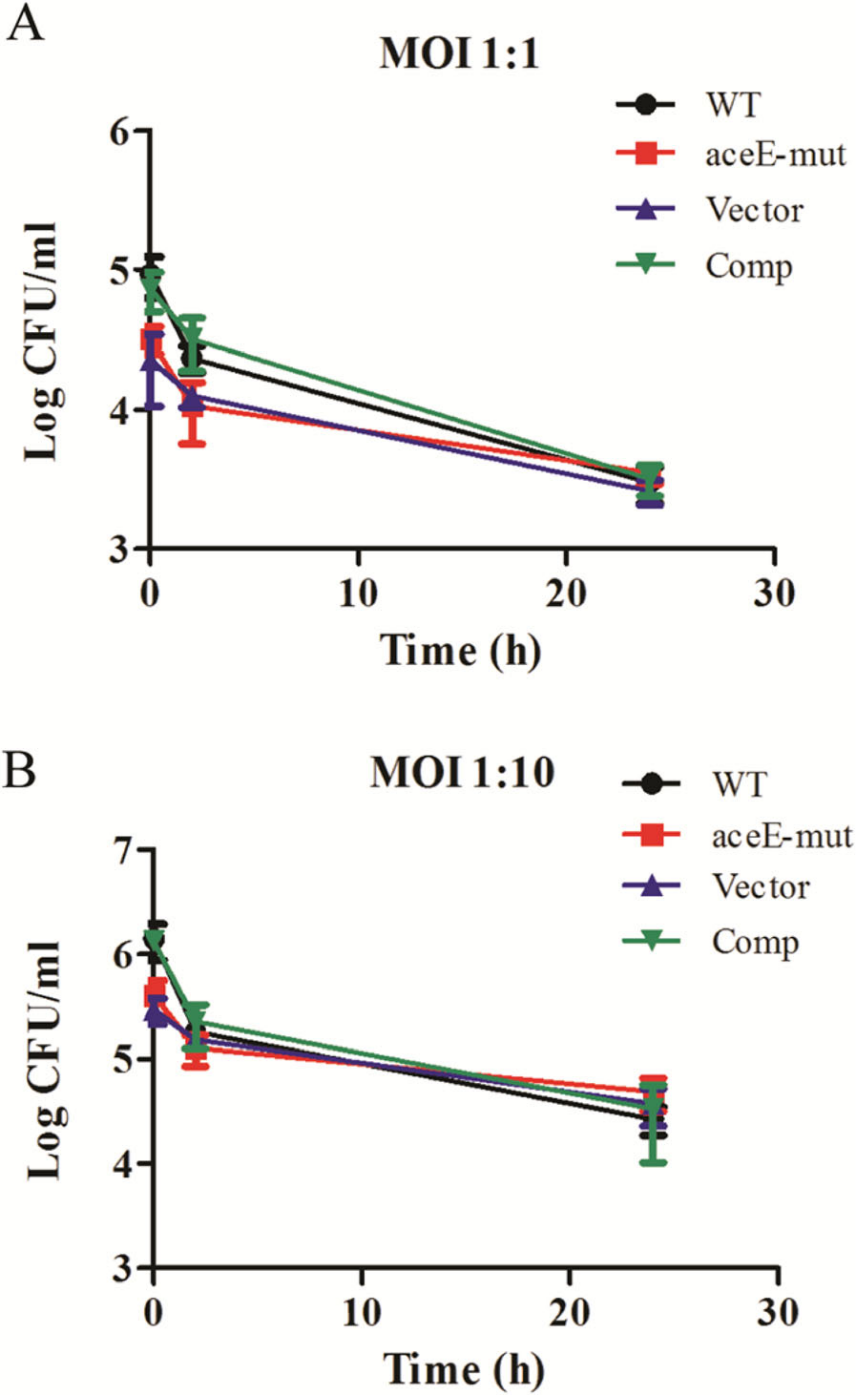
The effect of *aceE* deficiency on bacillus invasion of macrophages and intracellular growth. Data are depicted as mean values and standard deviations from 3 experiments performed in triplicates (*n* = 3). Statistical differences in bacterial loads were determined by One Way ANOVA test with Bonferroni correction, after conversion of CFU numbers in Log10 CFU values

The role of *aceE* in cell wall integrity and the *M. smegmatis* resistance against acidic environment suggests that it may also protect the bacilli from microbicidal activity of macrophages. Therefore, we investigated and compared the impact of *aceE* inactivation on invasion and intracellular survival of mutants with its corresponding complemented strain (Comp) and WT strain. As shown in Fig. 6, *aceE*-mut strains and its parent strain showed different abilities to invade THP-1 macrophages after 2 h of incubation. *aceE*-mut exhibited pronounced defects in entering the macrophages as compared to the WT and Comp strains. A significant difference between the mutant and WT strains of approximately 0.5 log unit was observed at t = 0 h post-infection (*P* < 0.05 at MOI = 1:1; *P* < 0.01 at MOI = 1:10). However, CFU of the mutant infection group was not lower than the WT infection group at t = 24 h.

### Disruption of *aceE* gene affected the mycolic acid composition

**Fig. 7:**
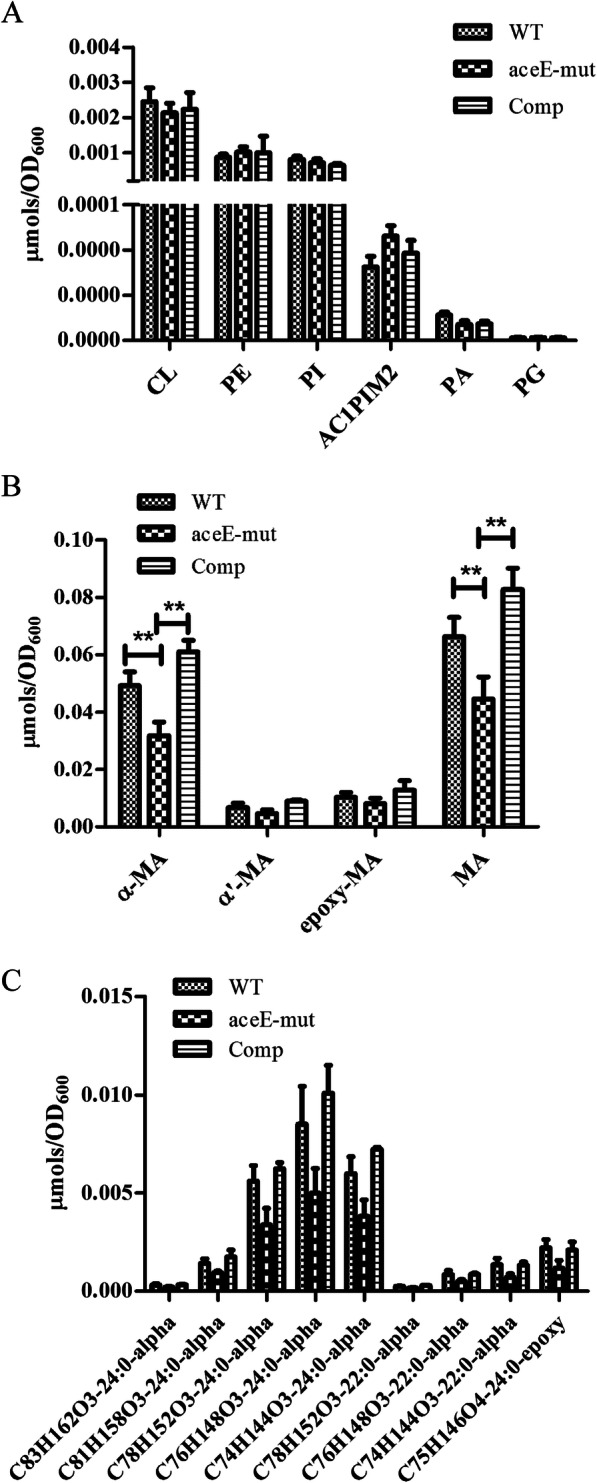
The effect of *aceE* deficiency on lipids composition of mycobacterium. Lipids were quantitated by LC-MS. **a**. The lipids composition other than mycolic acid of WT, *aceE*-mut and Comp strain; **b**. The mycolic acid composition of WT, *aceE*-mut and Comp strain. (**, *P* < 0.01); **c**. Only the components with significant difference in expression between *aceE-*mut and WT [and complementary (Comp)] strains were shown in the diagram (One Way ANOVA with Tukey’s HSD correction). Data are depicted as mean values and standard deviations from one experiment performed in quadruplicate (*n* = 4). CL, cardiolipins; PE, phosphatidylethanolamines; PI, phosphatidylinositols; AC1PIM2, Monoacylated Phosphatidylinositol Dimannoside; PA, phosphatidic acids; PG, phosphatidylglycerols; MA, mycolic acids

The mycolic acid (C_60_ ~ C_90_) composition was analyzed by HPLC using Mycobacteria Identification System. In contrast to WT strain, the mutant strain possessed higher proportion of short-chain mycolic acids but lower proportion of long-chain mycolic acids (Supplementary Fig. S2). The percentage of mycolic acid with equivalent carbon length of 5 to 8 in the mutant strain was significantly lower than the wild strain (*P* < 0.05), suggesting a potential role of *aceE* gene in mycolic acid metabolism. A further LC-MS-based systematic analysis of mycolic acid and lipids did not identify any obvious differences in the phospholipid and glycolipid compositions (*P* > 0.05) between the WT and *aceE*-mut strains (Fig. 7a), whereas certain kinds of α-mycolic acids were deficient in *aceE*-mut strains (*P* < 0.05) and these changes were fully restored upon complementation (Fig. 7b and c).

## Discussion

Transposon mutagenesis has been used extensively as a useful tool for studying gene function of mycobacteria. In this study, we identified an *aceE* gene mutant presenting deficient colony morphology on 7H10 agar using transposon mutagenesis method. Hence, it is reasonable to speculate defective cell wall biosynthesis in the mutant. Changes in the structure of cell envelope components may affect the normal physiological metabolic processes in mycobacteria including, transportation of antimicrobial agents across the plasma membrane and mycobacterial survival in stress conditions, etc.

Previous studies have shown that mycobacterial PDH genes (*aceE*, *dlaT* and *lpd*) are not only involved in pyruvate metabolism, but may also have more complex biological functions [12–16]. It has been observed that *dlaT* gene knockout significantly affects the in vitro growth of TB bacilli in the standard medium. The mutant is not only sensitive to reactive nitrogen intermediates but its virulence is also reduced in the infected mice. In addition, the study found that *dlaT* inhibitors can selectively kill non-self-replicating TB bacilli, suggesting that *dlaT* gene may be associated with latent TB infection in host cells [12, 14, 15]. Study also showed that the disruption of *lpd* gene in Mtb leads to decreased virulence with lower bacterial load in lung and other organs in infected mice. Other study revealed that the LPD is a component of branched chain ketones acid dehydrogenase, which takes part in succinyl CoA metabolism of amino acids such as valine (Val), isoleucine (Ile) [16].

However, the biological function of the E1 component of mycobacterial PDH remains poorly understood. In 2005, Tian et al., confirmed for the first time that *aceE* is the gene encoding the E1 component of PDH complex in Mtb [17]. In 2008, Li et al., found that the expression level of *aceE* gene in H37Rv was significantly higher than that of H37Ra during the course of macrophages infection [18]. This result is consistent with the results of gene chip analysis conducted by Manganelli et al., in 2001 [19]. Earlier studies had shown that H37Rv respiration is stronger than H37Ra. Although both, virulent H37Rv and the non-virulent H37Ra strains, rely on glycolysis and aerobic respiration for glucose metabolism, it is believed that glucose metabolism in H37Rv may be more dependent on the glycolytic pathway [20, 21]. Therefore, it is speculated that the up-regulated expression of *aceE* gene enables it to not only participate in the aerobic respiration as a component of PDH, but also guarantees successful glycolysis under the hypoxic conditions and thus provides excellent energy supply for growth of the virulent H37Rv strain. Another study showed that AceE component of PDH forms a four-component peroxidase system with DlaT/AhpD/AhpC, which assists the reductase action using pyruvate as a source of electrons [22], and thus protect the pathogen by participating in antioxidant of antinitraxidative defense. All of the above studies suggest that *aceE* gene plays an important role in Mtb metabolism in vitro and in vivo*.*

In the present study, we screened the random transposon mutants and found that the inactivation of *aceE* gene affects the colony morphology and biofilm formation in *M. smegmatis* which suggests its potential to affect the lipid metabolism and cell wall biosynthesis. The findings obtained in our study are in agreement with Viswanathan et al. [23]. Integrity of the cell wall has important biological significance for the in vitro and in vivo survival of bacteria. To further investigate the characteristics of the *aceE*-mut, series of assays were performed to compare the phenotypes of *aceE*-mut with the WT strains. We further analyzed the key composition of mycolic acids in each strain and identified altered composition in the *aceE*-mut which suggests that this gene may be involved in the metabolism of mycolic acid in mycobacteria. Therefore, it was necessary to thoroughly analyze the cell wall composition of both WT and *aceE*-mut strains and explore the role of *aceE* gene in the process of cell wall synthesis and metabolism. Comparative analysis of the lipid and mycolic acid profiles of the *M. smegmatis aceE*-mut, *M. smegmatis* WT and the Comp using LC-MS indicated that the metabolism of certain alpha-MA and epoxy-MA was deficient in *aceE*-mut, which demonstrated that the role of *aceE* was associated with mycolic acids, but not with other fatty acids found in *M. smegmatis*. In addition, we observed that the *aceE*-mut possess a distinct cell morphology and ultrastructural appearance compared with the WT strain when grown in broth culture, that may result from the inability of the mutants to synthesize certain kinds of alpha-MA. Thus, the inactivation of *aceE* also impacts bacterial physiology that ranges from reduced biofilm formation to changes in the cell morphology and cell wall thickness. Consistent with other studies, our study also found that the loss of long chain mycolic acids or oxygenated mycolic acids effects biofilm formation [24–26]. Previous study by Trivedi et al., showed that H37Rv are of marginally smaller size during biofilm formation, however, it was difficult to compare the differences in cell size between the *aceE*-mut and WT strains in our study because of the morphological differences between these two strains (*aceE*-mut strains is more slender than WT strains). Most importantly, *aceE*-mut was more susceptible to acidic environments than the parental *M. smegmatis*, suggesting plausible role of *aceE* gene in stress tolerance inside the host.

*aceE* gene is more readily expressed in the virulent Mtb H37Rv (than H37Ra) throughout the course of infection, that not only suggests its important role in the virulence, survival and persistence of Mtb, but also makes this gene a potential target for the development of newer vaccines and anti-TB drugs. In *M. smegmatis*, the full length of the MSMEG4323 (*aceE*) gene is 2790 bp. Using blast tools, *aceE* gene sequence of *M. smegmatis* was analyzed which highlighted its conservation in *M. smegmatis* and a similarity of 82% with the *aceE* gene in Mtb. As a model bacterium widely used in the study of functional genes of mycobacterium, *M. smegmatis* can also be used to investigate the potential role of *aceE* gene in mycobacterial cell wall biosynthesis. In the present study, the stress assay demonstrated that *aceE* gene helped mycobacteria to withstand acidic stress environment that also suggests its plausible role in stress tolerance inside the host. However, macrophage infection study showed that the inactivation of *aceE* gene in *M. smegmatis* does not affect bacterial proliferation in macrophages, but rather affects the ability of *M. smegmatis* to invade the macrophages. Therefore, we suggest that the *aceE* gene is a virulence factor of *M. smegmatis* that may be important in the initiation of infection in vivo. Since *M. smegmatis* does not have pathogenicity, the study using this bacterium model cannot reveal the possible function of *aceE* gene in Mtb pathogenesis. Therefore, the role of *aceE* gene in pathogenesis requires to be further explored using the virulent Mtb H37Rv strain.

## Conclusions

An *aceE* mutant *M. smegmatis* mc^2^155 strain selected from transposon library presented small, smooth morphology without jagged edges. Compared with its parental WT strain, *aceE*-mut lost the ability of growing aggregately as well as biofilm formation, and became more fragile to acidic stress. Additionally, alteration of the mycolic acid profile in *aceE*-mut may directly impact the overall cell wall morphology and acid sensitivity. All these changes of the mutant strain demonstrate that *aceE* gene inactivation reduces biosynthesis of α-MA, affects the integrity of mycobacterial cell wall, and decreases invasion of macrophage. Since acetyl-coA is an important precursor for the biosynthesis of mycolic acid, we hypothesized that the inactivation of *aceE* gene may lead to the restriction of acetyl-coA synthesis, thus affecting the biosynthesis of certain mycolic acids. Further study should be conducted to address this issue.

## Methods

### Strains, medium, condition

A transposon library was generated using *M. smegmatis* mc^2^155 as previously described [27, 28] and plated on 7H10 agar containing 20 mg/L kanamycin. Approximately, 1000 single colonies of variable sizes were randomly placed into 96 deep well plates containing 0.5 mL of Middlebrook 7H9 medium (BD Difco) containing kanamycin and grown at 37 °C. After 5 days of growth, cultures from each well were spotted on the Middlebrook 7H10 agar (BD Difco) plates, and more than 6 mutants were found for colony morphology defects. *E. coli* strain DH5α *pir 116* (kindly provided by Dr. Kaixia Mi) was used to identify the insertion site of the transposon mutant. *E. coli* strain Top10 (TransGen Biotech, China) was used to clone specific DNA fragments into pSMT3 plasmid (Table 1). When required, kanamycin (50 mg/L for *E. coli* and 20 mg/L for mycobacteria) and hygromycin (150 mg/L for *E. coli* and 75 mg/L for mycobacteria) were added to the growth medium.

**Table 1:** Strains and plasmids used in this study

Strain or plasmid	Relevant characteristic	Source or reference
Strains		
*M. smegmatis* mc^2^155	WT, ATCC19420	[29]
*aceE*-mut	mc^2^155 with *aceE*-mut disrupted by Himar1 transposon	This study
Vector	*aceE*-mut complemented with pSMT3-M plasmid	This study
Comp	*aceE*-mut complemented with pSMT3-*aceE*	This study
Plasmids		
Mar T7		[28]
pSMT3	Carries *hyg*^r^, *E. coli*-mycobacterial shuttle vector	[30]
pSMT3-*aceE*	*aceE* gene cloned under *hsp60* promoter in pSMT3-M vector	This study

### Transposon identification

To identify mutants with growth defects, genomic DNA was prepared from the selected transposon mutant. The genomic DNA was randomly digested with *BamH*I (Fermentas International Inc.) and then purified with a DNA extraction kit (Fermentas International Inc.). The purified DNA was ligated and transformed into DH5α *pir116* competent cells. The plasmids from the kanamycin-selected positive colony were isolated and sequenced with following primers: TLP1 5′-GCTGACCGCTTCCTCGTGCTTTA-3′; TLP2 5′-GCAGCGCATCGCCTTCTATC-3′.

### Construction of complemented strain of *aceE*-Mut

For complementation of *aceE*-mut strain, 2.79 kb full-length *aceE* gene (MSMEG_4323) from *M. smegmatis* was cloned into the mycobacterial shuttle vector pSMT3 [30] using NEBuilding pfu kit (New England Biolabs, Ipswich, MA), and pSMT3-*aceE* was generated (Table 1). The plasmid pSMT3-*aceE* was subsequently transformed into *aceE*-mut strain to generate the complemented strain i.e. Comp (Table 1). The transformants were selected on 7H10 agar plates, supplemented with 20 mg/L kanamycin and 75 mg/L hygromycin, followed by incubation at 37 °C for 3–4 days. The positively grown colonies were picked and identified by PCR-sequencing methods using following primers:

**Table:** 

aceE_S-FP1	5'-CGGGCTGCAGGAATTCGATTTGACCACCGAGTTCG-3'
aceE_S-RP1	5'-GACGGTATCGATAAGCTTGATTCAGGCGCTGCCGGTG-3'

### Colony morphology observation

To compare the colony sizes for different mycobacterial strains, log phase cultures were 10× serially diluted (1:10), grown on 7H10 medium at 37 °C and examined visually for any change. Photographs were taken after 3–4 days of incubation using stereo microscope (Leica MZ APO).

### Morphological observation by electron microscopy

Mycobacteria from log phase were harvested and washed with 0.1 M phosphate buffer (PBS). Cells were subsequently fixed using 2.5% glutaraldehyde. Post fixation was carried out in 1% osmic acid. Following several rinses with ddH_2_O, samples were dehydrated in a series of different concentrations of ethanol and 100% acetone. For transition solvent, resin: acetone (2:1) were used overnight. Epoxy resin-812 was used for 1 h for embedding. 90 nm sections were cut and stained with uranyl acetate and Reynold’s lead citrate (Ted Pella, Inc). After drying, transmission electron microscopic (TEM) images of the sections were taken using TEM-1400plus. The cell wall thickness was measured for each strain as follows: 100 mycobacteria in the visual field were randomly selected, the largest distance between the outer membrane and the inner membrane of each cell was measured and the data were statistically analyzed using One Way ANOVA with Bonferroni correction. For scanning electron microscopy (SEM), ethanol dehydrated samples were dried in freeze-drier and coated with 10 nm gold film using ion sputter. Scanning electron microscopic images were taken using HITACHI SU8010.

### Estimation of pellicle and biofilm formation

For pellicle formation assay, mycobacteria were inoculated in 4 ~ 5 mL of Middlebrook 7H9 medium without Tween-80 and grown at 37 °C without shaking. Biofilm formation was measured in M63-based liquid medium as previously described [31–34]. Biofilms of all three strains were grown in 96-well polystyrene plates or glass tubes containing M63-based liquid medium complemented with casein hydrolysate and glucose (without Tween-80), inoculated with 0.1% log phase culture, and incubated at 30 °C for 5–7 days under static conditions [33, 34]. The biofilm formation in each of the liquid cultures was qualitatively analyzed by photography and the images were processed using Adobe Photoshop CS5 software, and quantified with crystal violet staining, as previously described [31, 32].

### Growth profile of strains

The growth characteristics of *M. smegmatis* mc^2^155, *aceE*-mut, Vector (*aceE*-mut:Vector) and Comp (*aceE*-mut:*aceE*) strains were studied in neutral (pH 6.8) or acidified (pH 5.0, the pH was adjusted with hydrochloric acid) 7H9 medium. The cultures were inoculated with an initial optical density at 600 nm (OD_600_) of 0.01 and incubated at 37 °C with constant shaking at 200 rpm. OD_600_ was measured at specified time intervals and 10-fold serial dilutions were plated on 7H10 agar plates for colony forming unit (CFU) counts.

### Stress assays

To carry out in vitro stress studies, logarithmic phase *M. smegmatis* cultures (OD_600_ ~ 0.8) were harvested whereas diluted cultures were subjected to different stresses. For oxidative stress, *M. smegmatis* cultures at OD_600_ (~ 0.4) were exposed to hydrogen peroxide (H_2_O_2_, 0.1% or 1%) and CFU was determined after 24 h. For other stresses, *M. smegmatis* cultures, prepared as above, were adjusted to OD_600_ = 0.4 and NaNO_2_ (0.5% or 5%) or Sodium dodecyl sulfonate (SDS, 0.1% or 1%) was added. CFU was determined after 1 h with SDS and after 24 h with NaNO_2_.

### Antimicrobial susceptibility testing

Minimal Inhibitory Concentration (MIC) determination was performed by using the alamar blue microtiter assay as recommended in CLSI guidelines [6]. The antibiotics tested in the study include isoniazid (INH), rifampicin (RFP), ethambutol (EMB), ofloxacin (OFX), levofloxacin (LFX), moxifloxacin (MFX), amikacin (AMK) and capreomycin (CPM). The bacterial suspensions of 1.0 × 10^6^ CFU/well were seeded in 96-well plates in presence of antibiotics at concentrations 0.5, 1, 2, 4, 8, 16, 32, 64, 128, and 256 μg/mL and incubated at 37 °C for 2 days. Alamar blue dye was added in each well and the plates were re-incubated at 37 °C for 24 h. The color change from blue to pink indicated bacterial growth. The MIC was defined as the minimal concentration of the drug showed no color changes, which was the lowest concentration of drug that can inhibit the visible growth of the bacterium.

### Mycolic acid analysis using HPLC-Sherlock mycobacterium identification system

Bacterial cultures were collected for isolation of mycolic acids in the cell walls by extraction, saponification, and derivation according to instructions for the Sherlock Mycobacteria Identification System (SMIS; MIDI, Inc.). Mycolic acid composition of each sample was analyzed by SMIS using HPLC.

### Analysis of the composition of mycolic acids and lipids using LC-MS

Total lipids were extracted from the samples using an improved Bligh/Dyer extraction method (double extraction) and appropriate internal standards were added as previously described [16]. Analysis of mycolic acids and lipids was carried out using normal-phase LC-MS as previously described, with minor modification [35, 36]. The experiments were conducted with the help of Lipidall Technologies Company Limited (Changzhou, Jiangsu, China). Briefly, the Exion uplc-qtrap 6500 PLUS (Sciex) liquid-mass spectrometer was used for all of the experiments whereas the electric spray ionization (ESI) mode was used for all the analyses. The following conditions were used: curtain gas = 20, ion spray voltage =5500 V, temperature = 400 °C, ion source gas 1 = 35, and ion source gas 2 = 35. Phenomenex Luna 3-μm silica column (inner diameter 150 × 2.0 mm) was used to separate different kinds of polar lipids using mobile phase A (chloroform: methanol: ammonia 89.5:10:0.5) and B (chloroform: methanol: ammonia: water 55:39:0.5:5.5) using NP-HPLC. The gradient of mobile phase A was maintained for 5 min from 95%, then linearly decreased to 60% within 7 min and maintained for 4 min, and then it was further reduced to 30% and maintained for 15 min. Finally, the initial gradient was maintained for 5 min. Multiple reactions monitoring (MRM) conversion was established for the comparative analysis of various polar lipids and the signal intensity of each MRM value was normalized to an internal standard for quantitative comparisons.

### Macrophage infections

Bacteria at log phase was collected and washed with RPMI1640 before infection. Infection of THP-1 cell (ATCC TIB-202) was performed at a multiplicity of infection (MOI) of 10:1 and 1:1 (bacteria: macrophage), using the following conversion: an OD of 1 = 3 × 10^8^ CFU/mL. After 2 h of incubation at 37 °C, the extracellular mycobacteria were removed by three washings with 1 × PBS and RPMI1640 complete medium containing 100 μg/ml gentamycin was added to inhibit growth of exogenous mycobacteria in infected wells. At 0 h, 2 h and 24 h, infected macrophages were harvested and lysed with 0.1%Tween-80. Then the lysates were serially diluted with 0.05% Tween-80, and plated on 7H10 agar plates with or without the antibiotic. The plates were incubated at 37 °C until colonies could be counted.

### Statistical analysis

All statistical analyses were performed using SPSS statistics 21. Statistical differences were determined by One Way ANOVA with Bonferroni correction when comparing more than two groups. For mycolic acid and lipid quantification experiments, One Way ANOVA with Tukey’s HSD correction was used. Student t test was used to compare the statistical differences between two groups. Only *P* values < 0.05 were considered as statistically significant.

## Supplementary information

**Additional file 1: Figure S1.** Different growth of *M. smegmatis* mc^2^155 (WT) and *aceE*-mut after treatment with different chemical agents.

**Additional file 2: Figure S2.** The effect of *aceE* deficiency on mycolic acid composition in mycobacterium.

**Additional file 3: Table S1.** MICs for *M. smegmatis* mc^2^155 and *aceE*-mut with different antibiotics.

## Data Availability

All data generated or analysed during this study are included in this published article and its supplementary information files.

## Abbreviations

PDH: Pyruvate dehydrogenase complex
*aceE*-mut: *aceE*-deficient mutants
WT: Wild-type
MA: Mycolic acid
Mtb: *Mycobacterium tuberculosis*
anti-TB: Anti-tuberculosis
MIC: Minimal Inhibitory Concentration
SMIS: Sherlock Mycobacteria Identification System
ESI: Electric spray ionization
MRM: Multiple reactions monitoring
CFU: Colonies forming unit
SDS: Sodium dodecyl sulfonate
ECL: The Equivalent Carbon Length of the mycolic acid
H_2_O_2_: Hydrogen peroxide
INH: Isoniazid
RFP: Rifampicin
EMB: Ethambutol
OFX: Ofloxacin
LFX: Levofloxacin
MFX: Moxifloxacin
AMK: Amikacin
CPM: Capreomycin
TEM: Transmission electron microscopy
SEM: Scanning electron microscopy
CL: Cardiolipins
PE: Phosphatidylethanolamines
PI: Phosphatidylinositols
AC1PIM2: Monoacylated Phosphatidylinositol Dimannoside
PA: Phosphatidic acids
PG: Phosphatidylglycerols
