# Supplementary figures and images for: The aceE involves in mycolic acid synthesis and biofilm formation in Mycobacterium smegmatis
Source: BMC Microbiol. 2020 Aug 18;20:259. doi: 10.1186/s12866-020-01940-2 (PMC7437000; doi:10.1186/s12866-020-01940-2)

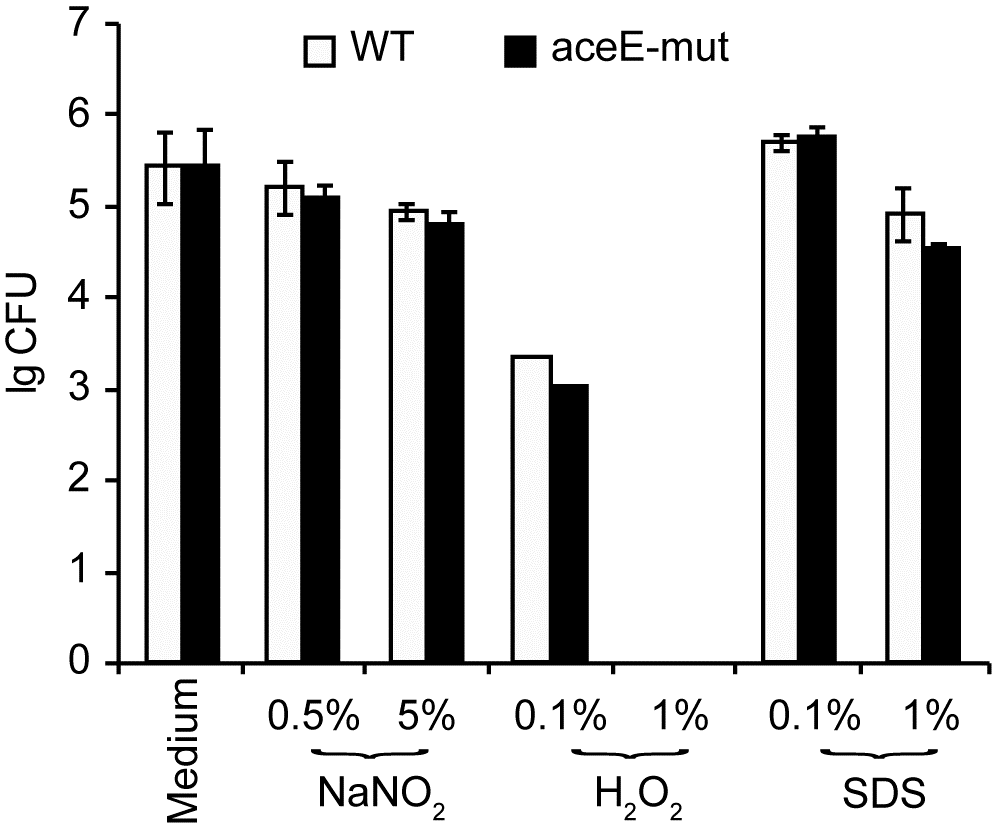


Supplementary Figure 1.

Supplement: Supplementary file 1 — Additional file 1: Figure S1. Different growth of M. smegmatis mc2155 (WT) and aceE-mut after treatment with different chemical agents. [file 12866_2020_1940_MOESM1_ESM.docx]

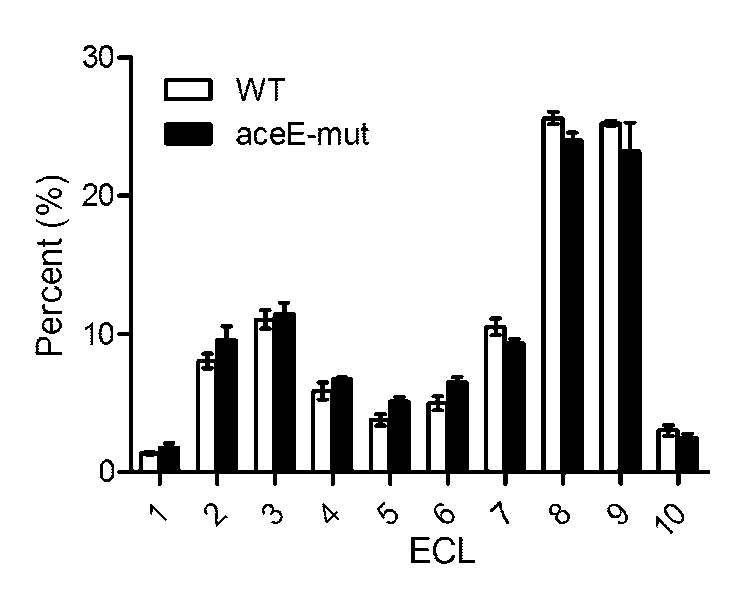


Supplementary Figure 2.

Supplement: Supplementary file 2 — Additional file 2: Figure S2. The effect of aceE deficiency on mycolic acid composition in mycobacterium. [file 12866_2020_1940_MOESM2_ESM.docx]
