# Supplementary material for: The aceE involves in mycolic acid synthesis and biofilm formation in Mycobacterium smegmatis
Source: BMC Microbiol. 2020 Aug 18;20:259. doi: 10.1186/s12866-020-01940-2 (PMC7437000; doi:10.1186/s12866-020-01940-2)
Supplement: Supplementary file 3 — Additional file 3: Table S1. MICs for M. smegmatis mc2155 and aceE-mut with different antibiotics. [file 12866_2020_1940_MOESM3_ESM.docx]

Supplementary. Table 1. MICs for *M. smegmatis* mc^2^155 and *aceE*-mut with different antibiotics

| Antibiotic(s) | MIC (μg/ml) for | | | MIC ratio (WT/*aceE*-mut) |
| --- | --- | --- | --- | --- |
|  | WT | *aceE*-mut | Comp |  |
| INH | >256 | >256 | >256 | 1 |
| RFP | 128 | 128 | 128 | 1 |
| EMB | 8 | 8 | 8 | 1 |
| OFX | 16 | 16 | 16 | 1 |
| LFX | 8 | 8 | 8 | 1 |
| MOX | 4 | 4 | 4 | 1 |
| AMK | 128 | 128 | 128 | 1 |
| CPM | 128 | 128 | 128 | 1 |
